# Evaluation of Highway Pavement Structural Conditions Based on Measured Crack Morphology by 3D GPR and Finite Element Modeling

**DOI:** 10.3390/ma18143336

**Published:** 2025-07-16

**Authors:** Zhonglu Cao, Dianguang Cao, Haolei Chang, Yaoguo Fu, Xiyuan Shen, Weiping Huang, Huiping Wang, Wanlu Bao, Chao Feng, Zheng Tong, Xiaopeng Lin, Weiguang Zhang

**Affiliations:** 1 Tianjin Port Engineering Institute Co., Ltd., of CCCC First Harbor Engineering Co., Ltd., Tianjin 300222, China; caozhonglu@126.com; 2 CCCC First Harbor Engineering Co., Ltd., Tianjin 300461, China; caodianguang@163.com; 3 School of Transportation, Southeast University, Nanjing 211189, China; 230238938@seu.edu.cn (H.C.); 220233324@seu.edu.cn (X.S.); linxiaopeng@seu.edu.cn (X.L.); wgzhang@seu.edu.cn (W.Z.); 4 Chongqing Airport Group Co., Ltd., Chongqing 401120, China; fu_yaoguo@163.com; 5 Ningxia Transportation Construction Co., Ltd., Yinchuan 750002, China; huanzou43149@163.com (W.H.); jtqg2035@163.com (H.W.); baowanlu0519@163.com (W.B.); fengchao202305@163.com (C.F.)

**Keywords:** road engineering, structural cracks, three-dimensional radar, three-dimensional simulation, evaluation index, maintenance suggestion

## Abstract

Structural cracks are internal distresses that cannot be observed from pavement surfaces. However, the existing evaluation methods for asphalt pavement structures lack the consideration of these cracks, which are crucial for accurate pavement assessment and effective maintenance planning. This study develops a novel framework combining a three-dimensional (3D) ground penetrating radar (GPR) and finite element modeling (FEM) to evaluate the severity of structural cracks. First, the size and depth development of structural cracks on a four-layer asphalt pavement were determined using the 3D GPR. Then, the range of influence of the structural crack on structural bearing capacity was analyzed based on 3D FEM simulation model. Structural cracks have a distance-dependent diminishing influence on the deflection in the horizontal direction, with the most pronounced effects within a 20-cm width zone surrounding the cracks. Finally, two indices have been proposed: the pavement structural crack index (PSCI) to assess the depth of crack damage and the structural crack reflection ratio (SCRR) to evaluate surface reflection. Besides, PSCI and SCRR are used to classify the severities of structural cracks: none, low, and high. The threshold between none/low damage is a structural crack damage rate of 0.19%, and the threshold between low/high damage is 0.663%. An experiment on a 132-km expressway indicated that the proposed method achieved 94.4% accuracy via coring. The results also demonstrate the strong correlation between PSCI and pavement deflection (R^2^ = 0.92), supporting performance-based maintenance strategies. The results also demonstrate the correlation between structural and surface cracks, with 65.8% of the cracked sections having both structural and surface cracks.

## 1. Introduction

By the end of 2023, the total expressway length in China had exceeded 183,600 km, with approximately 50% of the roads having a service life of 10 years or more. International engineering and research experience indicate that the 8-year service time is a critical stage for the initiation of structural cracks, such as reflective and fatigue cracks on expressways, owing to the combined influence of traffic loads, climate conditions, and material degradation, while the 10-year service time is key for the further development of structural cracks [1]. Structural cracks refer to internal damage existing within pavement structures (including surface course, base, or subbase layers) that may penetrate layer interfaces, while surface cracks are shallow fissures confined to the pavement surface [2]. Structural cracks are not only crucial for estimating the remaining service life of asphalt pavements but also serve as the basis for maintenance planning. Accurate evaluation of structural crack characteristics is an urgent need for the development of the industry.

“Highway Technical Condition Evaluation Standards” (JTG 5210-2018) [3], issued by the Ministry of Transport of China, established the Pavement Condition Index (PCI) to measure the effects of asphalt pavement distress, including structural cracks. PCI classifies asphalt pavement distresses into 11 categories, with a focus on surface condition assessment. For evaluating structural bearing capacity, the PSSI index, based on deflection, is used. The advantage of this approach lies in its intuitive concept and ease of calculation. However, its drawbacks include low sensitivity to different types of internal structural damage, an inability to effectively assess interlayer adhesion conditions, and a limited capability in determining the size of internal structural cracks.

In terms of structural damage assessment, Guangdong Province in China has conducted extensive statistical analysis on the quantity of structural damages at different layer positions and developed an evaluation model for internal structural conditions, which considered structural looseness, high moisture content, and delamination [2]. The research on the Jinggang’ao Expressway proposed a geometric morphology index of deflection basins to indirectly characterize structural cracks, enabling the identification of internal cracks [4]. Ground penetrating radar (GPR) has been extensively adopted worldwide for non-destructive evaluation of transport infrastructures, with highway applications focusing on layer thickness measurement and internal distress detection (e.g., structural cracks and voids), while GPR was also used for the detection of airport runway internal distress [5]. In Jiangsu Province, a pavement structural damage condition index (IPCI) based on ground penetrating radar (GPR) has been developed for expressway pavement condition assessment, assigning delamination, poor interlayer bonding, and looseness, with different weights [6]. A study on the Shanghai–Chengdu Expressway established a pavement structural damage assessment model, categorizing excessive porosity and interlayer discontinuity into five severity levels: mild, moderate, severe, very severe, and extremely severe [7]. The research on the structural health of the Foshan Expressway pavement established a three-dimensional damage rate, enabling three-dimensional analysis of pavement structural damage [8].

Researchers have made significant progress in evaluating structural damage in expressway asphalt pavements. However, current studies still face several key challenges: (1) There is a lack of sufficient consideration of structural cracks in structural damage evaluation, even though structural cracks are a significant cause of pavement deterioration and repair needs. (2) Existing research on structural crack evaluation lacks targeted damage identification, with high subjectivity in assessment, insufficient validation of indicators, and no established connection with existing evaluation systems. (3) The correlation between structural cracks and surface cracks is not clearly defined. To address these issues, this study utilizes three-dimensional ground penetrating radar (3D GPR) to extract the characteristics of structural cracks. It also employs a finite element simulation model to investigate the impact of different structural cracks on the overall structural bearing capacity of the highway. An evaluation index for structural cracks is established, and the analytic hierarchy process (AHP) and entropy weight method (EWM) are used to calculate the weight coefficients of structural cracks and surface cracks. The spatial correspondence between structural cracks and surface cracks is clarified, providing a basis for evaluating structural cracks and formulating maintenance measures.

In summary, significant progress has been made in evaluating structural damage in expressway asphalt pavements. Still, several critical issues remain: (1) Structural cracks, as a significant factor contributing to pavement deterioration and repair needs, have not been sufficiently considered in current pavement distress detection, evaluation, and maintenance decision-making research. (2) Existing studies on pavement structural cracks lack a targeted quantitative evaluation system and indicators, exhibiting problems such as intense subjectivity in assessment, insufficient validation of the impact of indicators on pavement bearing capacity, and disconnection from established evaluation systems. (3) The correlation between structural cracks and surface cracks remains unclear.

To address these issues, this study employs 3D GPR technology to extract various types of structural cracks with different shapes and locations within the pavement structure. Combined with finite element simulation models, the influence mechanisms of these different types of structural cracks on the overall bearing capacity (i.e., deflection) of the pavement are analyzed, thereby establishing quantitative evaluation indicators and thresholds for the severity of structural cracks. Finally, the AHP and EWM are applied to calculate the weight coefficients of structural cracks and surface cracks, clarifying their spatial correspondence. This analysis proposes a pavement condition classification that incorporates quantitative indicators of structural cracks and formulates maintenance and treatment measures for each classified condition.

## 2. Data Collection

Based on the 2022 pavement maintenance project of Shandong Province’s expressways, a 132-km section of the RiLan Expressway (G1511), from K191+000 to K258+000, including both directions of traffic and a single lane, was selected for testing. Data on surface conditions, structural bearing capacity, and structural damage of the asphalt pavement were collected. This section, opened in 2002, is a four-lane, dual-carriageway with a semi-rigid base and asphalt pavement type. A typical structure of this section is shown in Table 1.

**Figure 1:**
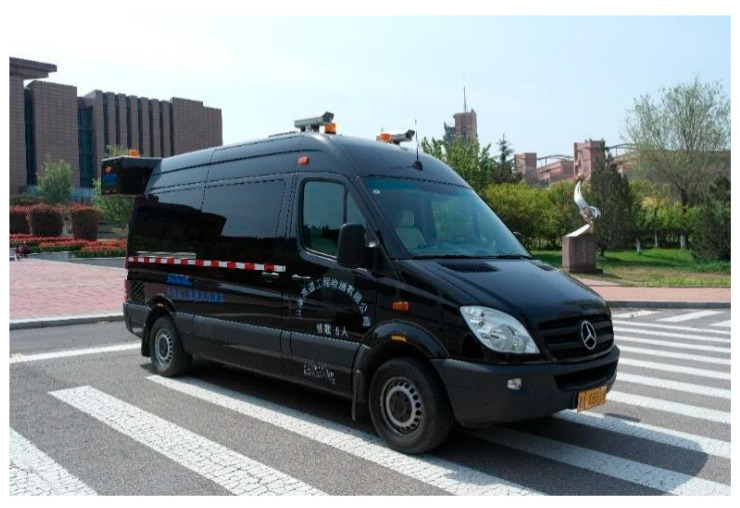
Road data comprehensive inspection vehicle.

In the research section, surface condition data were collected using an integrated intelligent survey vehicle equipped with multi-index detection modules. The inspection vehicle employs a GPS/DMI integrated positioning system. By combining hardware instruments such as line-scan cameras, line lasers, laser rangefinders, accelerometers, and laser imaging-based rutting instruments with corresponding control software HTD160/800 V1.0, it collects fundamental data including the road damage index (PCI), road roughness index (RQI), road rutting depth index (RDI), road surface skid resistance index (PWI), road bump index (PBI), and forward-view landscape imagery. Figure 1 shows the road data comprehensive inspection vehicle. Table 2 summarizes the various surface distresses, where transverse cracks and longitudinal cracks are the primary forms of distress, accounting for a total of 95.7%.

Data were collected using a falling weight deflectometer (FWD), and the average values and ranges of deflection and PSSI are summarized in Table 3. The average deflection value of the pavement is 17.65 × 0.01 mm, and the average PSSI value is 93.355.

Structural damage data were collected using a 3D GPR (IDS-900MHz) with a ground-coupled antenna for signal transmission and reception [9]. Table 4 summarizes the statistics of various structural damages, where the number of structural cracks represents the largest proportion (59.5%) and is the predominant form of distress.

## 3. Correlation Between Structural Cracks and Surface Cracks

### 3.1. Structural Crack Identification

**Figure 2:**
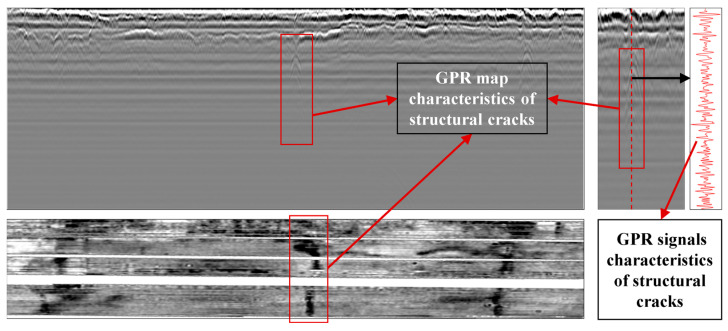
3D radar map characteristics of structural cracks.

Based on the 3D GPR data, the presence of structural cracks in a road section can be determined, and information such as the crack’s location, size, and structural layer position can be identified. Figure 2 shows typical features of a three-dimensional GPR profile. Structural cracks exhibit distinct echo characteristics in both the vertical and horizontal planes of the radar profile, which can be used to infer crack information. In the vertical section, the radar signal at both the top and bottom of the crack forms a downward-opening hyperbola. The reflection energy at the top is stronger, and the vertex of the hyperbola at the top is brighter in grayscale compared to the vertex at the bottom. When the crack is narrow, the wings of the hyperbola are narrower; when the crack is wider, the wings are broader. In the horizontal plane, the crack appears as a narrow curved region, and the degree of curvature can help determine the crack’s development trend between layers [10,11]. The GPR profile in both vertical and horizontal sections provides high precision, allowing the software to directly determine the crack’s direction, structural layer position, and its length and depth. Structural crack width is often on the millimeter scale, much smaller than the length and depth, and the accuracy of width identification using radar is relatively lower.

The total length of the radar detection section is long, and manual identification efficiency is low. Therefore, the radar automatic identification software developed by the research team was utilized for profile identification and the extraction of structural damage features [12]. This software locates targets using both horizontal and vertical line positioning, and simultaneously displays the 3D profile features of the target signal. It automatically determines the echo intensity and waveform of structural cracks, and computes the true length and depth directions of the cracks. Based on the two-way travel time method, the calculation formulas for crack depth are shown in Equations (1)–(3) as

(1) $$h_{cu}^{\prime }=\frac{1}{2}v_{u}t\prime_{1}=\frac{1}{2}\frac{c}{\sqrt{\varepsilon \prime_{u}}}t\prime_{1}$$

(2) $$h\prime_{cm}=h_{u}+\frac{1}{2}v_{m}\left(t\prime_{2}-t_{u}\right)=h_{u}+\frac{1}{2}\frac{c}{\sqrt{\varepsilon \prime_{m}}}\left(t\prime_{2}-\frac{2h_{u}\sqrt{\varepsilon \prime_{u}}}{c}\right)$$

(3) $$h\prime_{cl}=h_{u}+h_{m}+\frac{1}{2}v_{i}\left(t\prime_{3}-t_{u}-t_{m}\right)=h_{u}+h_{m}+\frac{1}{2}\frac{c}{\sqrt{\varepsilon \prime_{l}}}\left(t\prime_{3}-\frac{2h_{u}\sqrt{\varepsilon \prime_{u}}}{c}-\frac{2h_{m}\sqrt{\varepsilon \prime_{m}}}{c}\right)$$

where the calculated depths of cracks located at the top of the upper, middle, and lower layers are denoted as $h\prime_{cu}$, $h\prime_{cm}$, and $h\prime_{cl}$ (m); the actual burial depths of the cracks at the top of the upper, middle, and lower layers are denoted as $h_{cu}$, $h_{cm}$, and $h_{cl}$ (m), respectively; the average relative permittivity of the upper, middle, and lower asphalt layers inhomogeneous is $\varepsilon \prime_{u}$, $\varepsilon \prime_{m}$, and $\varepsilon \prime_{l}$, while the actual relative permittivity at the measurement line is $\varepsilon_{u}$, $\varepsilon_{m}$, and $\varepsilon_{l}$, respectively; the two-way travel time of the electromagnetic wave in the upper, middle, and lower layers is $t\prime_{u}$, $t\prime_{m}$, and $t_{l}\prime (s)$, respectively; c (m/s) is the speed of light, and the wave speed in the upper, middle, and lower layers ($v_{u}$, $v_{m}$, $v_{l}$) (m/s) is calculated by $v=\frac{c}{\sqrt{\varepsilon }}$. The crack length is significantly larger than the crack depth, making it easier to extract after profile recognition.

The structural cracks are classified based on their development layers: categorized into cracks that exist only in the base layer, cracks that exist only in the surface layer, and cracks that exist in both the surface and base layers. The quantity, length, depth, and width range of each type of structural crack are shown in Table 5. Among them, structural cracks that exist only in the base layer account for 82.9% and are not observable by manual inspection, making them more difficult to detect. Structural cracks that exist only in the surface layer make up 3.6%, with relatively mild severity. Structural cracks that penetrate both the surface and base layers account for 13.5%, with these cracks being longer and more severely damaged.

### 3.2. Field Verification of Structural Crack Morphology

Core sampling and milling were conducted on sections with structural cracks to verify the accuracy of the 3D GPR in detecting structural cracks. A total of 72 core samples were taken, and the results are shown in Table 6. Among these, 94.4% of the structural cracks caused significant damage to the surface or base layer, while only 5.6% had a minimal impact on the structural integrity.

**Figure 3:**
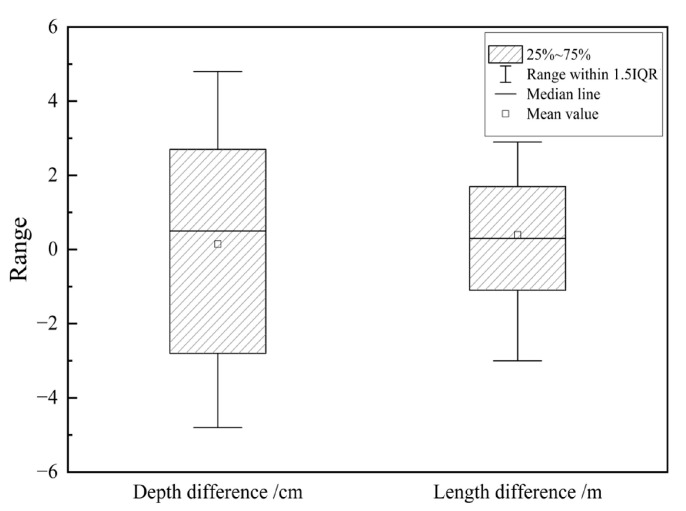
Verification of crack detection results of ground penetrating radar based on core samples.

Figure 3 shows the relationship between the actual structural crack depth values obtained from core samples, the actual structural crack length values obtained from milling, and the detection values from the radar spectra. The crack depth error is concentrated within the range of −3 cm to 3 cm, with a mean of 0 cm. The crack length error is concentrated within the range of −1 m to 2 m, with a mean of 0.2 m, confirming the accuracy of the ground penetrating radar detection.

**Figure 4:**
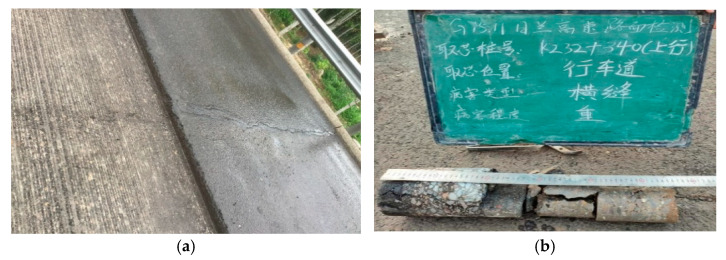

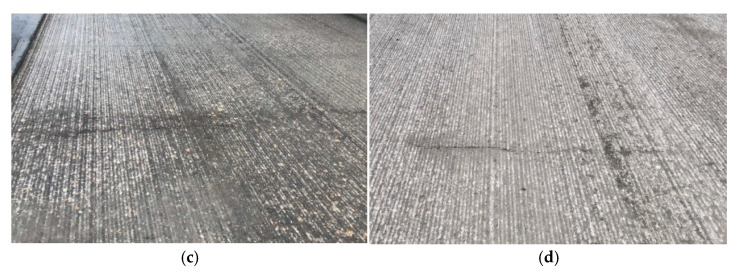
Transverse crack K232 + 340 up. (**a**) Transverse surface cracks in the field, (**b**) Core sampling in the field (The text in the image is: Rilan Expressway Pavement Inspection; Core Sampling Stake Number K232 + 340 up; Location: Travel lane; Defect Type: Transverse crack; Severity Level: Severe), (**c**) Cracks after milling the upper surface layer, and (**d**) Cracks after milling the lower surface layer.

Figure 4 presents the core sampling and milling results for sections with severe structural cracks. Figure 4a shows the surface condition of the section, where transverse cracks span both the travel lane and the passing lane. Previous maintenance measures, such as crack sealing, were applied; however, the treatment proved ineffective. Figure 4b presents the core sampling results near the hard shoulder, where both the surface layer and the upper base layer exhibit severe cracking, and the lower layer and base layer show significant fragmentation with poor interlayer bonding. Figure 4c,d show the milling results layer by layer, revealing multiple structural cracks in the surface layer.

### 3.3. Matching of Structural Cracks and Surface Cracks

**Figure 5:**
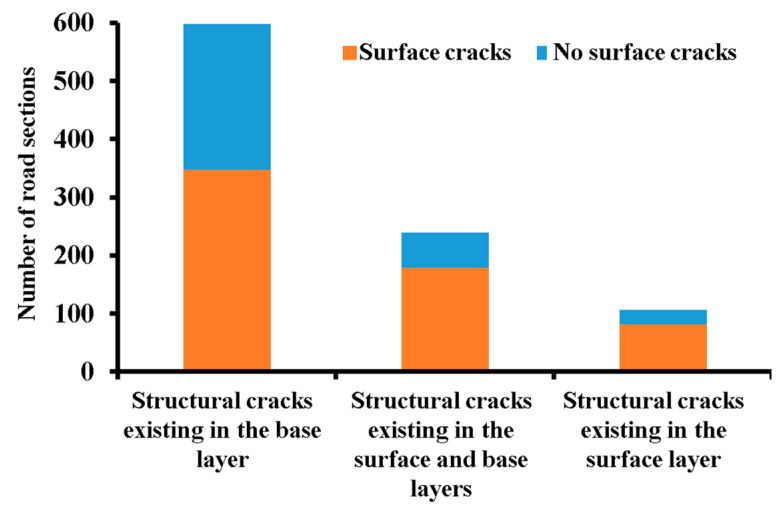
Statistics of the number of structural cracks and surface cracks.

In the previous research [13], the research team theoretically analyzed and confirmed the presence of base layer cracks, which increased the probability of surface cracks. This study, using actual measurement data, investigates whether structural cracks and surface cracks occur on the same sections of the road. As shown in Figure 5, there are 588 sections with structural cracks in the base layer, and 58% of these sections also have surface cracks, with a pile number difference of less than 7 m between the two types of cracks. For the 218 sections with structural cracks in both the surface and the base layers, 74.5% of these sections also exhibit surface cracks, with a pile number difference of less than 7 m. For the 86 sections with structural cracks only in the surface layer, 76.4% of these sections also have surface cracks, with a pile number difference of less than 7 m. The occurrence of structural cracks and surface cracks in the same sections shows a clear correspondence, with 64.3% of the crack sections having both structural and surface cracks. Therefore, there is a significant match between structural cracks and surface cracks.

## 4. The Impact of Structural Cracks on the Load-Bearing Capacity

To analyze the impact of different types of structural cracks on highway deflection, a finite element 3D model was established using ABAQUS 2022 software [14]. The overall dimensions, structural layer parameters, boundary conditions, and load distribution of the model were determined based on actual data. Structural cracks with various characteristics were designed, and calculations were performed.

### 4.1. Viscoelastic Constitutive Model of Asphalt Mixture

An asphalt mixture is a composite material consisting of aggregates, asphalt binder, and mineral fillers, whose properties are dependent on temperature, frequency, and loading conditions. With decreasing temperature and increasing loading frequency, the modulus of the asphalt mixture transitions from low to high values. Under high-stress (or high-strain) loading conditions, the material exhibits pronounced nonlinear behavior and irrecoverable plastic characteristics. Consequently, asphalt mixture represents a typical thermo–viscoelastic–viscoplastic material, where the total strain can be decomposed into recoverable viscoelastic strain and irrecoverable viscoplastic strain components as

(4) $$\varepsilon =\varepsilon_{ve}+\varepsilon_{vp}$$

where $\varepsilon_{ve}$ is the viscoelastic strain of the asphalt mixture under load; and $\varepsilon_{vp}$ is the viscoplastic strain of asphalt mixture under a load.

The constitutive model of the asphalt mixture can be divided into two parts: the viscoelastic response and the viscoplastic response. This study focuses on the viscoelastic strain behavior of asphalt pavements under impact loading, and therefore, the constitutive model only analyzes the viscoelastic response part. Under standard loading conditions with small strains (ε<70~100 με), the deformation can gradually recover after the load is removed. Additionally, the mechanical properties are temperature-dependent. The linear viscoelastic integral-type constitutive relation between stress and strain is given as [15].

(5) $$\sigma_{ij}=\frac{1}{3}\sigma_{kk}\delta_{ij}+S_{ij}\in =\delta_{ij}\int_{0}^{t}K\left(\zeta \left(t\right)-\zeta \left(\tau \right)\right)\frac{\partial \left(\varepsilon_{kk}-\varepsilon^{T}\right)}{\partial \tau }d\tau +2\int_{0}^{t}G\left(\zeta \left(t\right)-\zeta \left(\tau \right)\right)\frac{de_{ij}}{d\tau }d\tau$$

where $\sigma_{ij}$ (Pa), $\sigma_{kk}$ (Pa), and $\delta_{ij}$ (Pa) are the stress tensor, volumetric stress tensor, and deviatoric stress tensor, respectively; $\varepsilon_{kk}$, $e_{ij}$, and $\varepsilon^{T}$ are the volumetric strain tensor, deviatoric strain tensor, and thermal strain tensor, respectively; $K\left(t\right)$ (Pa) and $G\left(t\right)$ (Pa) are the bulk relaxation modulus and shear relaxation modulus of the material, respectively; $\zeta \left(t\right)$ is the environmental adjustment function of the material; and $\delta_{ij}$ is the Kronecker delta function.

The bulk relaxation modulus and shear relaxation modulus can be expressed in terms of the Young’s relaxation modulus and Poisson’s ratio as

(6) $$K\left(t\right)=\frac{E\left(t\right)}{3\left(1-2v\right)}$$

(7) $$G\left(t\right)=\frac{E\left(t\right)}{2\left(1+v\right)}$$

where $E\left(t\right)$ (Pa) represents the Young’s relaxation modulus, and v is the material’s Poisson’s ratio. According to the Chinese highway asphalt pavement design specifications, the Poisson’s ratio for asphalt mixtures is specified as 0.25.

The relaxation modulus is a key indicator for characterizing viscoelastic mechanical behavior. Based on the generalized Maxwell model, it is expressed in the form of a Prony series as

(8) $$E\left(t\right)=E_{\infty }\sum_{i=1}^{n}E_{i}e^{\frac{-t}{\tau_{i}}}$$

where $E_{\infty }$ (Pa) represents the material’s permanent elastic modulus; $E_{i}$ (Pa) and $t\left(s\right)$ are the relaxation modulus and relaxation time under different relaxation spectra, respectively, reflecting the relaxation characteristics of various components of the material.

### 4.2. Thickness of Road Structural Layers and Material Parameters

Based on the pavement structure model of the Rilan Expressway G1511, Table 7 presents the thickness of each pavement structural layer, along with the corresponding material parameters, including elastic modulus, Poisson’s ratio, and density. Table 8 lists the viscoelastic parameters, which were calculated from dynamic modulus tests conducted in the laboratory [16]. The Poisson’s ratio for the surface layer, base layer, and subbase layer is 0.25, while the Poisson’s ratio for the subgrade is taken as 0.40. All parameters were determined based on laboratory test measurements.

### 4.3. Loading and Boundary Conditions

The loading model and boundary conditions are set according to the FWD detection principle, with the deflection values used to represent the strength of the pavement structure’s bearing capacity.

Based on “Highway Subgrade and Pavement Field Test Procedures” (JTG 3450-2019) [17] and field experience in test point layout, the test point configuration is determined as shown in Table 9.

The applied load can be simplified as a uniformly distributed load within a circular area of radius 0.15 m, following a half-wave sinusoidal distribution [18]. The peak load is 0.7 MPa, with a duration of 30 ms.

The road is considered to have an infinite horizontal dimension. To better match the actual loading conditions, the bottom of the model is fully constrained, the top is free, and symmetric boundary conditions are applied to the four lateral faces of the model.

### 4.4. Mesh Division

**Figure 6:**
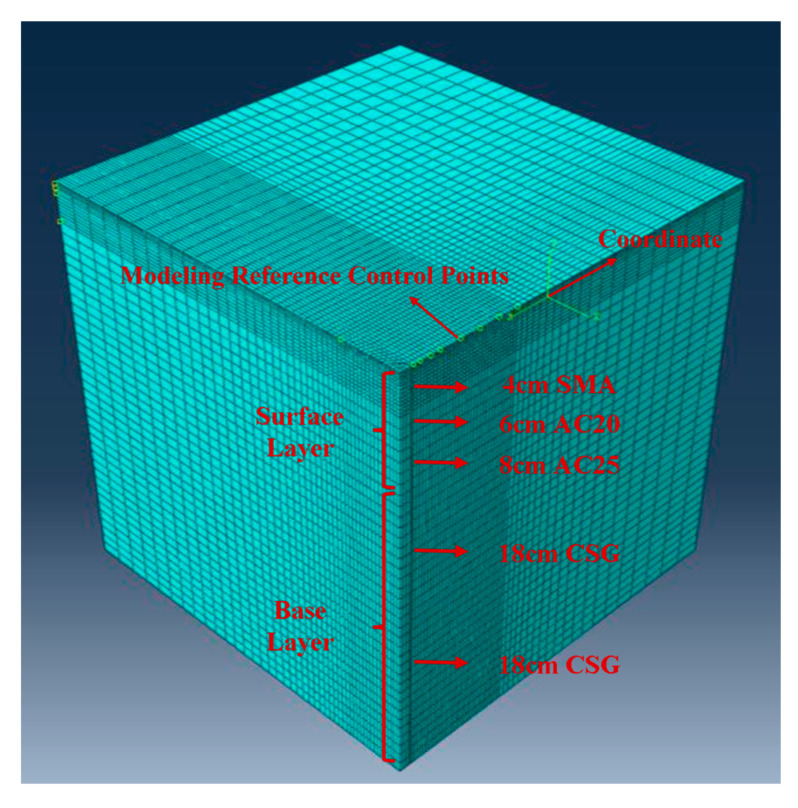
Pavement finite element model.

The ideal highway model should be infinitely large in the longitudinal and depth directions, and have a finite width in the lateral direction. In finite element simulations, appropriate 3D dimensions need to be chosen to ensure computational efficiency while minimizing the impact of model size on the simulation results. Based on prior research [19], a quarter model with dimensions of 6m × 6m × 6m in the longitudinal (X), lateral (Z), and depth (Y) directions is selected for this study. The global mesh size is set to 0.15 m, with finer mesh refinement of 0.05 m along the direction of sensor measurement points. The eight-node linear hexahedral elements (C3D8R type) are selected, and a compatible display integration method is used [20], as shown in Figure 6. Reduced integration is applied for the calculation.

### 4.5. Structural Crack Morphologies

The crack morphological feature parameters are shown in Table 10. A total of 112 three-dimensional models with structural cracks (referred to as crack pavement models) and one three-dimensional model without cracks (referred to as the intact pavement model) were established and computed.

### 4.6. Model Validation

**Figure 7:**
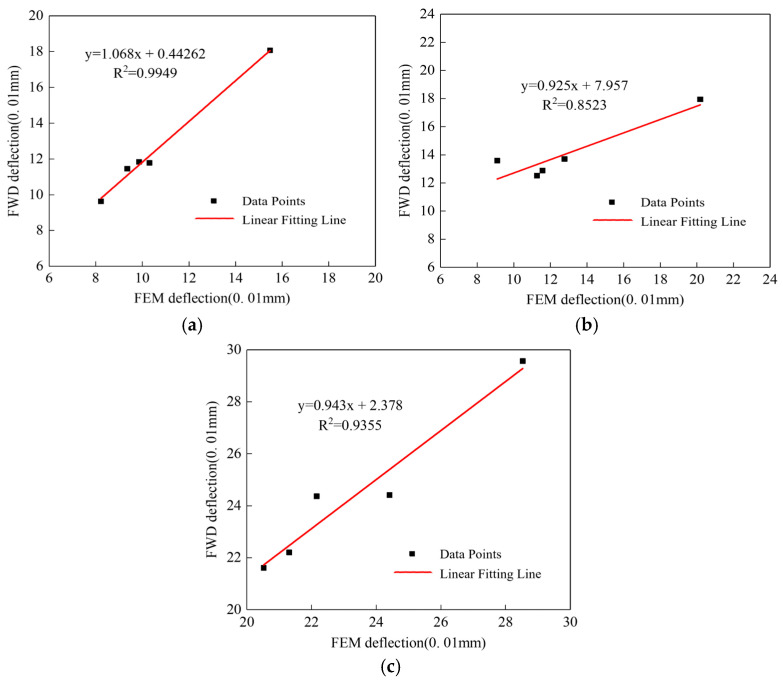
Correlation analysis between FWD measurements and finite element model deflection responses for the tested pavement sections: (**a**) 3-mm width, 3.75-m length structural crack section, and (**b**) 5-mm width, 3.75-m length structural crack section, and (**c**) 10-mm width, 7.5-m length structural crack section.

The FWD was used to evaluate the structural bearing capacity, while finite element modeling simulated pavement deflection in cracked sections. To ensure the accuracy of the evaluation model for subsequent research and to jointly validate the findings, a correlation analysis was conducted between FWD-measured deflection data and finite element simulation results. Figure 7 presents the correlation analyses for: (a) a 3-mm wide, 3.75-m long structural crack section, (b) a 5-mm wide, 3.75-m long structural crack section, and (c) a 10-mm wide, 7.5-m long structural crack section.

## 5. Research on Structural Crack Evaluation Criteria

This study establishes a structural crack evaluation index to assess the degree of structural damage based on the morphological information of structural cracks. It also introduces a structural crack reflection coefficient to provide a comprehensive evaluation of both structural and surface cracks. Furthermore, a weighting calculation method for various parameters is developed based on the AHP and the EWM.

### 5.1. Structural Crack Evaluation Index

The actual length of structural cracks multiplied by the affected width gives the affected area. The ratio of the affected area to the pavement survey area is the structural crack rate (SCR). A larger SCR value indicates a more severe level of damage. SCR is then converted into a percentage scale to obtain the pavement structure crack index (PSCI), which represents the severity of structural crack damage. The calculation process is shown in Equations (9) and (10).

(9) $$SCR=100\times \frac{\sum_{k=1}^{k_{0}}\omega_{k}A_{k}}{A}$$

(10) $$PSCI=100-a_{0}SCR^{a_{1}}$$

where $A_{k}$ represents the accumulated affected area (m^2^) of the k-th type of structural crack; A represents the pavement survey area (m^2^), calculated as 100 m in length by a single lane width of 3.75 m; k=1,2,3 corresponds to structural cracks that exist only in the base layer, only in the surface layer, or simultaneously in both the base and surface layers; $\omega_{k}$ is the weight coefficient for the k-th type of structural crack, with the calculation and verification process described in Section 5.3; $a_{0}$ and $a_{1}$ are model parameters, with values 25.120 and 0.555, respectively, as detailed in Section 6.3 [5,21]. Based on the structural crack evaluation, the pavement structure condition is classified into three levels: excellent, good, and medium, as shown in Table 11.

### 5.2. Structural Crack Reflection Ratio

To establish the mathematical relationship between structural cracks and surface cracks, and to quantify the effect of structural cracks on the initiation and propagation of surface cracks, a comprehensive indicator, the structural crack reflect ratio (SCRR), has been introduced. SCRR is the ratio of surface crack damage rate to structural crack damage rate, representing the severity of structural cracks relative to surface cracks. CR is the ratio of the surface crack area to the pavement survey area, and SCR is consistent with Section 4.1. The calculation is shown in Equations (11) and (12).

(11) $$CR=100\times \frac{A_{0}}{A}$$

(12) $$SCRR=\frac{CR}{SCR}$$

where CR (crack rate) represents the surface crack damage rate; $A_{0}$ is the total area (m^2^) of surface transverse and longitudinal cracks; the meanings of A and SCR are consistent with those in Equation (11).

A threshold value of SCRR = 1 is used to classify the comprehensive evaluation of structural and surface cracks, as shown in Table 12.

### 5.3. Composite Weight Assignment Based on AHP and EWM

The AHP [22] decomposes the structural crack evaluation indicators into different levels, with values assigned based on expert experience. The EWM [23], supported by actual structural crack measurement data, calculates the entropy weight for each indicator, which is then modified to obtain the final weights.

This study adopts a composite weighting method that combines the AHP and EWM [22] for weight calculation. By integrating expert experience and engineering practice, the importance of each indicator is assessed. Real data is used to ensure the accuracy and stability of the weights, thereby reducing the subjectivity that may arise from AHP and mitigating the potential discrepancy between the calculation results and engineering expectations that can occur due to reliance on measured data. The formula for calculating the combined weight $\omega_{i}$ using the AHP-EWM composite weighting method is as

(13) $$\omega_{i}=\alpha \omega_{1}+\left(1-\alpha \right)\omega_{2}$$

where $\omega_{1}$ represents the subjective weight obtained from the AHP; $\omega_{2}$ is the objective weight obtained from EWM; and α is the adjustment coefficient w.r.t the combined weight.

To minimize the sum of the squared deviations between the combined weight $\omega_{i}$ and the subjective weight $\omega_{1}$, as well as between $\omega_{i}$ and the objective weight $\omega_{2}$; the adjustment coefficient α=0.5 is chosen accordingly. After obtaining $\omega_{i}$, the median value is selected, and then it is scaled logarithmically with 1 as the baseline to obtain the final combined weight $\omega_{i}\prime$.

### 5.4. Grading Threshoalds Based on the Fuzzy C-Means (FCM) Algorithm

The Fuzzy C-Means (FCM) algorithm is the most widely used clustering method, allowing each data point to belong to multiple clusters with different degrees of membership. Through data collection, normalization, threshold calculation, and result analysis and verification, the structural crack evaluation index is divided into N categories, and the grading thresholds are obtained. In the calculation process, structural crack size data, deflection data, and core extraction data are normalized. The number of clusters N is defined and assigned values of 2, 3, and 4, respectively, and the cluster centers and membership degrees are calculated, as shown in Equations (14) and (15) as follows:

(14) $$c_{i}=\frac{\sum_{i=1}^{n}u_{ij}^{m}x_{i}}{\sum_{i=1}^{n}u_{ij}^{m}}$$

(15) $$u_{ij}=\frac{1}{\sum_{k=1}^{C}{\left(\frac{x_{i}-c_{j}}{x_{i}-c_{k}}\right)}^{\frac{2}{m-1}}}$$

where $c_{j}$ represents the center of the j-th cluster; n is the total number of data points; $m\left(\geq 1\right)$ is the weighted index that controls the fuzziness or the degree of overlap in the clusters; $u_{ij}$ is the degree of membership of data point $x_{i}$ (with normalized texture ratio) in the j-th cluster. In the first iteration; $u_{ij}^{m}$ is randomly assigned. In the second iteration; $u_{ij}^{m}$ is calculated based on the cluster center; and $u_{ij}$ indicates the degree of membership of data point $x_{i}$ in the j-th cluster.

The key to the FCM algorithm is to minimize the value of the objective function. The objective function measures the quality of the partition by comparing the distance of a single data point $x_{i}$ to the current candidate cluster center $c_{j}$ with the distance to other candidate cluster centers. The objective function is the optimization function for calculating the weighted sum of squared errors within the cluster (WGSS), as shown in Equation (16).

(16) $$J_{m}=\sum_{i=1}^{n}\sum_{j=1}^{c}u_{ij}^{m}\left\|{\left.x_{i}-c_{j}\right\|}^{2}\right.$$

where $\left\|{\left.x_{i}-c_{j}\right\|}^{2}\right.$ represents the Euclidean distance from the i-th data point to the j-th cluster center.

## 6. Results Analysis and Field Validation

In this study, we sought to analyze the impact of structural cracks on deflection, determine the weight coefficient of structural cracks based on measured data, and validate the accuracy of the evaluation indicators presented in this study using field inspection, coring, and milling results. Based on the structural crack evaluation indicators, we provide optimization suggestions for maintenance plans.

### 6.1. The Impact Width of Structural Cracks on Deflection

**Figure 8:**
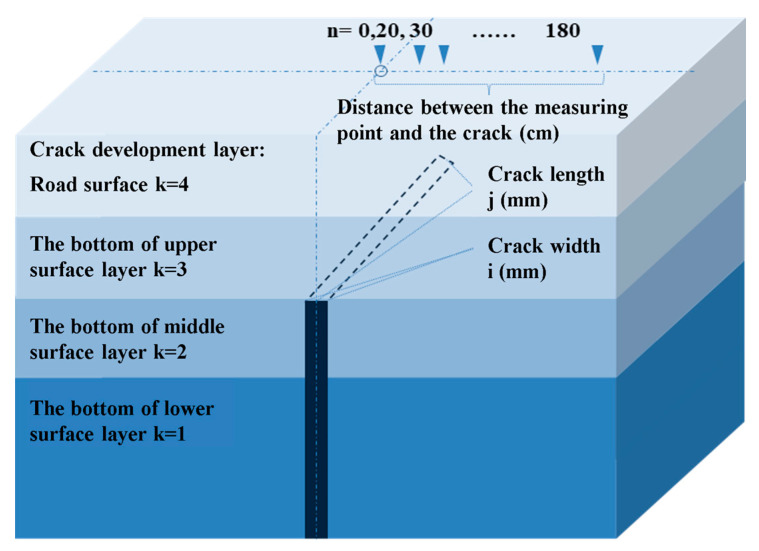
Deflection calculation diagram.

The deflection ratio $R_{i-j-k-n}$ in Equation (17) is defined as the ratio of the deflection at the location of the structural crack to the deflection at a location without structural cracks, used to evaluate the effects of structural cracks on the bearing capacity.

(17) $$R_{i-j-k-n}=\frac{D_{i-j-k-n}}{D_{0-n}}$$

where the deflection at the location of the structural crack is $D_{i-j-k-n}$, as shown in Figure 8, and the deflection at a location without structural cracks is $D_{0-n}$. The parameters i, j, k, and n represent the length, width, development level, and the distance from the measurement point to the midpoint of the crack, respectively.

**Figure 9:**
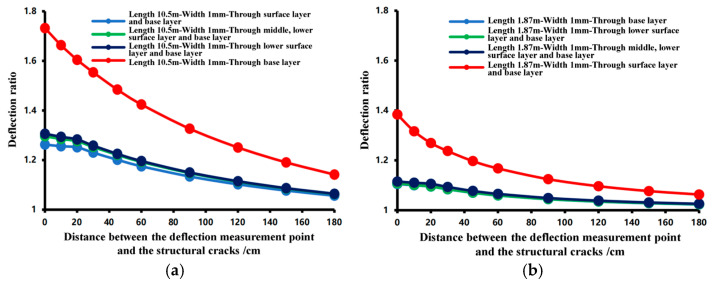
Bending ratio curve based on simulation calculation. (**a**) Structural crack: length 10.5 m, width 15 mm, and (**b**) Structural crack: length 1.87 m, width 1 mm.

**Figure 10:**
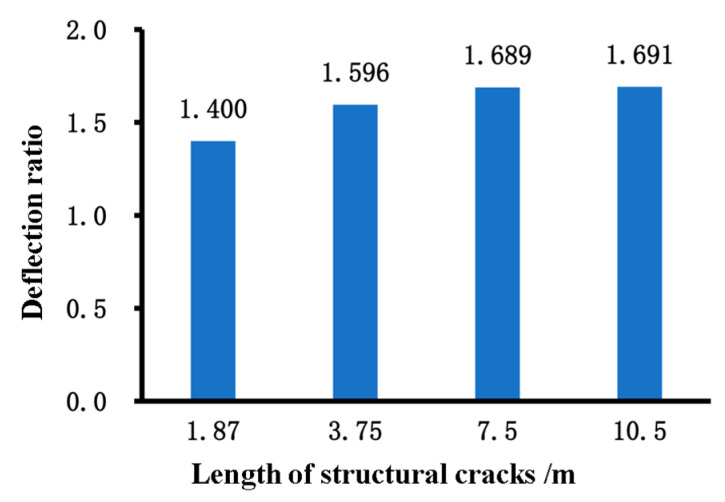
Midpoint deflection ratio of structural cracks of different lengths.

**Figure 11:**
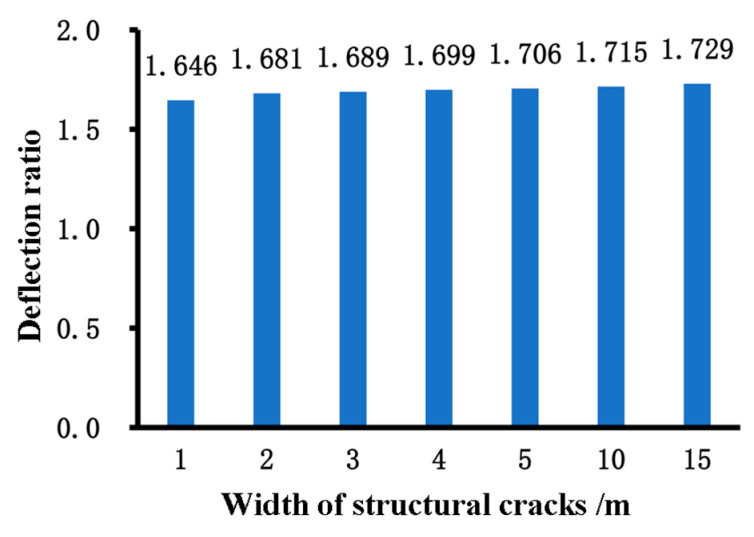
Midpoint deflection ratio of structural cracks of different widths.

Figure 9, Figure 10 and Figure 11 present the calculation results. Figure 9a compares the deflection ratio curves of a structural crack with a length of 10.5 m and a width of 15 mm at different structural layers. The following observations can be made as follows.

(1) When the structural crack exists in the base layer, between the base layer and the underlying layer, or between the base layer and the middle underlying layer, the deflection ratio curves are similar, indicating that the impact of the structural crack on deflection is nearly the same in these cases. The deflection ratio decreases as the distance from the crack increases. When n ≤ 20 cm, the deflection ratio is relatively high, and when n > 20 cm, the rate of decrease in the deflection ratio becomes significant, eventually approaching 1.
(2) When the structural crack penetrates through the base surface layer and develops to the road surface, the midpoint deflection ratio (n = 0) increases by 32% compared to the three curves in (1), indicating that the crack has the most significant impact on deflection. For n ≤ 20 cm, the deflection ratio is high, and for n > 20 cm, the deflection ratio is less than 80% of the midpoint deflection ratio.

Figure 9a,b compares the analysis results of structural cracks located at the same structural layer but with different crack sizes. As the crack length and width decrease, the trend of the curves remains consistent. The impact on deflection is greatest when n ≤ 20 cm, and the impact diminishes when n > 20 cm. Therefore, 20 cm is taken as the impact width of the structural crack.

Figure 10 compares the midpoint deflection ratio for structural cracks with different lengths, a width of 5 cm, and extending through the base layer and the middle underlying layer. When the crack length is less than 7.5 m, the ratio increases gradually, with a maximum increase of 20.7%, indicating that the impact of the crack length on deflection increases gradually. When the crack length is greater than or equal to 7.5 m, the ratio reaches its maximum value, and the impact on deflection is most significant when the crack length exceeds two traffic lanes.

Figure 11 compares the midpoint deflection ratio for structural cracks with different widths, a length of 7.5 m, and extending through the base layer and the middle underlying layer. As the crack width increases, the midpoint deflection ratio gradually increases, with a maximum increase of only 5%. This suggests that the impact of crack width on pavement deflection is relatively weak.

### 6.2. The Weight Coefficient of the Structural Crack Evaluation Indicator

Based on the combination of the AHP and EWM, the weight coefficients are calculated using measured data. The calculation scenarios include structural cracks that exist only in the base layer, in both the surface layer and the base layer, and in the surface layer.

Through field consultations and surveys with maintenance practitioners, senior engineers from maintenance companies, and university experts, an analytic hierarchy process (AHP) judgment matrix was established to obtain the AHP weights $\omega_{1}$. Using information entropy, the entropy weights of each indicator were calculated, and after correction, the entropy weight method yielded the weights $\omega_{2}$. The combined weight coefficients $\omega_{i}\prime$ were obtained using Equation (12), and the calculation results are shown in Table 13.

### 6.3. Grading Thresholds for Structural Crack Evaluation Indicators

**Figure 12:**
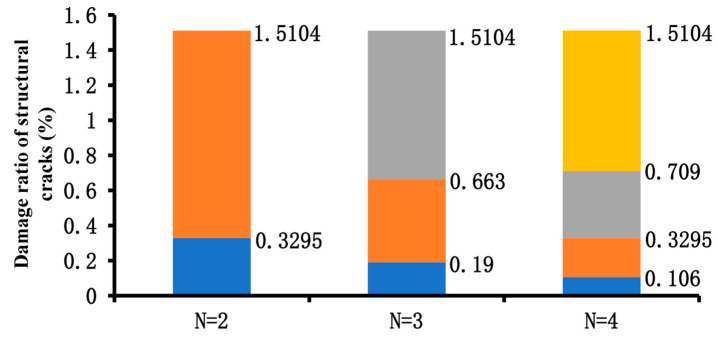
Summary of the final thresholds.

Based on the Fuzzy C-Means (FCM) algorithm, Table 14 presents the clustering results of normalized data under different classification scenarios. When N = 2, the pavement sections are divided into two groups: one with structural cracks and one without. The results for N = 3 and N = 4 are also shown, demonstrating clear and effective differentiation based on the clustering results for N = and N = 4. The final determined thresholds are summarized in Figure 12.

**Figure 13:**
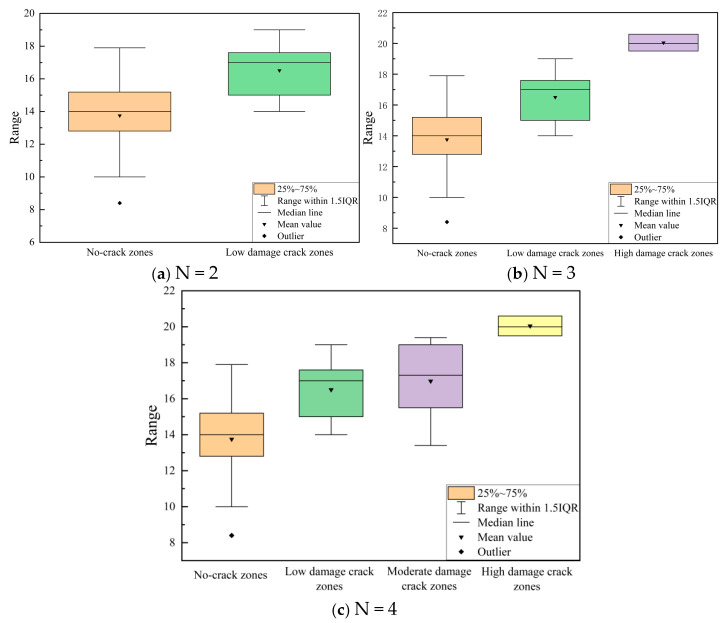
Effect of failure rate of structural cracks on pavement midpoint deflection.

Figure 13 illustrates the impact of structural crack damage rate on the midpoint deflection of the pavement. The interquartile range (IQR) quantifies the statistical dispersion of the middle 50% of the data by calculating the difference between the 75th (Q3) and 25th (Q1) percentiles. In boxplots, the height of the box visually represents the data, with the median marked as an internal line, while whiskers typically extend to 1.5 times the interquartile range (IQR) to identify potential outliers. Statistical *t*-tests (for N = 2) and analysis of variance (for N = 3 and N = 4) were used to analyze whether there were significant differences between the groups. The results show that, in (a), the midpoint deflection in areas with structural cracks is greater than in areas without structural cracks. Panel (b) displays a clear gradient of midpoint deflection, with higher damage zones corresponding to higher midpoint deflection values, while the no-crack zone shows the lowest midpoint deflection values. Statistical analysis also reveals that, in (c), the differences in midpoint deflection values between the no-crack zone, low-damage zone, and moderate-damage zone are minimal.

**Figure 14:**
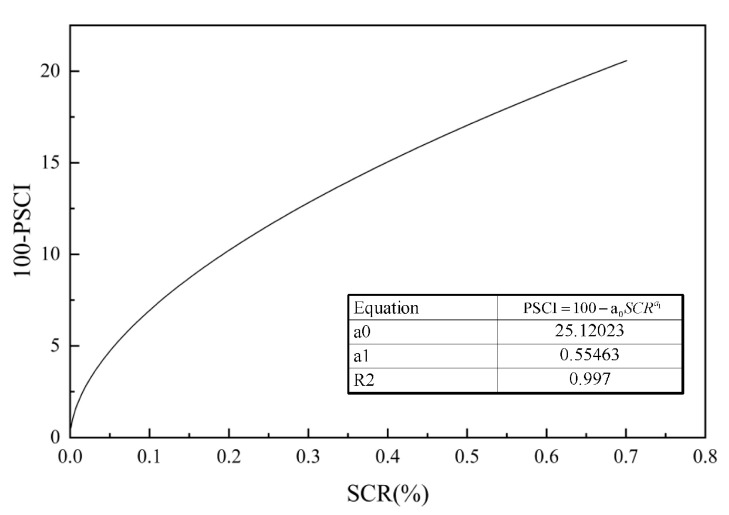
Nonlinear fitting results of PSCI and SCR.

Based on the calculation results and deflection validation, structural cracks are best categorized into three groups: no structural cracks, low-level structural cracks, and high-level structural cracks. This is because when divided into two groups, the number of sections without structural cracks is too large, resulting in poor validation outcomes. When divided into four groups, the differentiation between low-level and moderate-level damage is insufficient. The final determined structural crack damage rate thresholds are as follows: 0.19% to distinguish between no structural cracks and moderate damage, and 0.663% to distinguish between mild and high-level damage. These thresholds correspond to PSCI (structural crack index) values of 90 and 80, respectively. Nonlinear fitting using Origin 2017 software yielded the parameters a_0_ = 25.120 and a_1_ = 0.555, as shown in Figure 14.

### 6.4. Field Validation

Based on the measured data, evaluation indicators are calculated for each 100-m unit, including the PSSI specified in the Highway Technical Condition Evaluation Standard (JTG 5210-2018), the invisible crack rate (ICR) developed by other researchers [21], as well as the structural crack evaluation index (PSCI) and structural crack reflection ratio (SCRR) developed in this study.

The evaluation indicator calculation results are shown in Table 15. The PSSI value is 93.355, indicating that the overall structural strength of the pavement sections is excellent, with 95.9% of the sections rated as excellent and 4.1% rated as good or below. The ICR value is 0.278%, suggesting that the overall invisible crack rate of the pavement is excellent, with 82.7% of the sections rated as excellent and 17.3% rated as good or below. Based on the PSCI evaluation index thresholds, the average PSCI value is 92.391, with 75.5% of the sections rated as excellent and 24.5% rated as good or below. According to the SCRR evaluation index thresholds, the SCRR value is 0.340, indicating that the area of structural crack damage is greater than the surface crack damage area. Specifically, 19.8% of the sections have structural cracks that are not fully reflected in the upper layers, but rather exist only in the base layer or surface layer.

The PSSI is calculated based on deflection and primarily represents the pavement structure’s ability to resist deformation, without considering structural cracks. The ICR also takes structural crack damage into account, but assigns the same weight to cracks in different layers, overlooking the varying impact of various types of structural cracks on structural damage. The PSCI developed in this study considers structural cracks. For pavement sections with good structural strength but severe cracks, they are classified as “good or below,” indicating the need for maintenance and repair. Without intervention, these cracks could further develop or allow moisture ingress, leading to significant damage.

**Figure 15:**
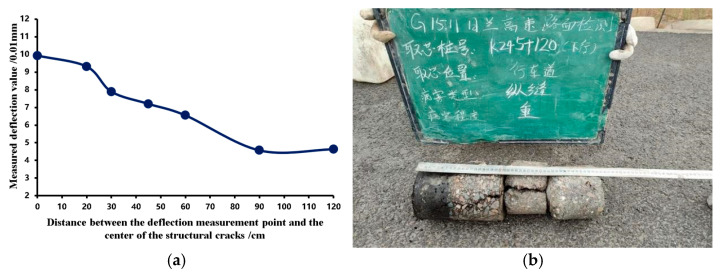
Core sample results and bending basin data at ascending K230+328. (**a**) FWD measured deflection basin data, (**b**) Core sample results (The text in the image is: Rilan Expressway Pavement Inspection; Core Sampling Stake Number K245 + 120 down; Location: Travel lane; Defect Type: Longitudinal Crack; Severity Level: Severe).

Figure 15 shows the deflection basin data for a structural crack and the field core sample results. The measured midpoint deflection value is 106.8 mm × 0.01 mm, yielding a PSSI of 99.97, which indicates excellent structural strength. However, the core sample results reveal structural cracks between the surface and base layers, causing severe interlayer damage.

Through the above analysis, the structural crack damage index and structural crack reflection ratio provide a reasonable, accurate, and more stringent evaluation of the pavement structural condition, demonstrating good applicability.

### 6.5. Maintenance Recommendations Based on Structural Crack Evaluation Method

Based on the research findings, the following maintenance recommendations for structural cracks are proposed:

(1) Evaluate Pavement Structural Condition and Classify Sections

The evaluation of pavement structural condition considers four indicators: PSCI, SCRR, PCI, and PSSI, as defined in the relevant standards. Indicators with weaker correlations to structural condition and those that generally perform well on highways (according to other standards) are excluded from the evaluation. Pavement sections are classified based on this evaluation, with the classification criteria shown in Table 16. The reference range for the evaluation indicator standard values is provided in Table 17.

(2) Maintenance Measure Classification and Maintenance Recommendations

The maintenance measures are classified according to the different pavement conditions, as shown in Table 18. The maintenance measures for localized crack treatment include polymer injection, self-adhesive sealing tapes, geogrid installation, and localized patching [24,25].

Class I Sections: It is recommended to either not perform maintenance or to apply P1-level maintenance measures.

Class II Sections: The treatment recommendations are as follows:

If there are upcoming maintenance plans, apply P2-level maintenance measures.

If there are no upcoming maintenance plans, no treatment should be performed, but structural crack development should be monitored and included in the next maintenance plan.

Class III Sections: Apply P2-level maintenance measures.
Class IV Sections: P3-level maintenance measures can be applied.
Class V Sections: P4-level maintenance measures can be applied.
Class VI Sections: P5-level maintenance measures can be applied.
Class VII Sections: P6-level maintenance measures can be applied.

**Figure 16:**
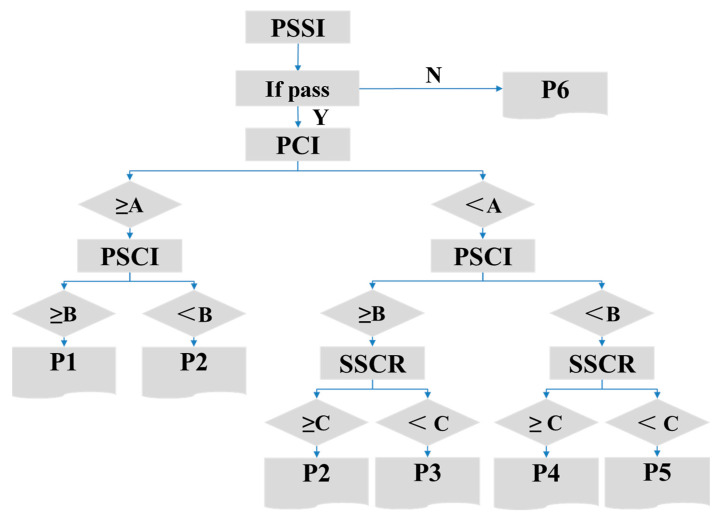
Maintenance decision tree based on structural crack evaluation method.

Based on the influence of structural crack length and width on pavement deflection as discussed in Section 6.2, when the structural crack length exceeds the width of two lanes, the crack should be treated as a priority, and the maintenance level should be upgraded by one level [26,27,28]. Figure 16 illustrates the maintenance decision tree based on the structural crack evaluation method.

## 7. Conclusions

Based on 3D GPR data and FEM simulation, a structural crack evaluation index for asphalt pavements on highways was developed. This index considers both crack depth positions and reflection ratios, enabling a high-precision evaluation of the crack condition. Targeted maintenance recommendations were then proposed based on the form of the structural cracks. The conclusions are as follows:

(1) The impact of structural cracks on deflection decreases in the horizontal direction with increasing distance, with the most significant effect observed within a 20-cm width range of the cracks. In the depth direction, the impact increases with the depth of the crack penetration.
(2) The PSC for evaluating the degree of structural crack damage and the SCRR for assessing the proportion of cracks reflected on the surface were established. It was confirmed that there is an association between structural cracks and surface cracks (65.8% of cracked sections have both types of cracks at the same location).
(3) In the validation section, structural crack damage is preferably categorized into three groups: none, low, and high structural crack damage. The threshold between nonstructural and low-structural crack damage is set at 0.19% structural crack damage rate, while the distinction between low and high structural crack damage is set at 0.663%. While the two-group classification resulted in an excessive number of regions being categorized as nonstructural crack damage, the four-group classification showed insufficient distinction between the low and moderate structural crack damage groups, leading to poor validation results.
(4) Maintenance recommendations for structural cracks were proposed, including a classification of road conditions based on PSCI, SCRR, PCI, and PSSI. Maintenance and treatment measures were then formulated for each classified road condition.
(5) The thresholds for quantifying and classifying structural damage severity based on crack damage rate, along with the pavement structural evaluation system proposed in this study, were established for specific aggregate gradations and aggregate/bitumen sources. These thresholds and evaluation criteria may not apply to other mixtures, such as open-graded friction courses. Future research may employ the methodology presented herein to develop customized thresholds for assessing structural crack damage in specific asphalt mixtures. This approach could also be adapted for other pavement investigations, such as determining severity thresholds for asphalt pavement rutting and raveling.

## Figures and Tables

**Table 1:** Typical pavement structure.

Structural Layer	Material Type	Thickness/cm
Surface layer	SMA-13	4
	AK-16	4
	AC-20	5
	AC-25	6
Base layer	Cement stabilized crushed stone	2 × 18
Subbase layer	Cement stabilized gravel	18

**Table 2:** Amount of various pavement surface distresses.

Surface Distress Type	Quantity (Occurrences)
Transverse cracks	2141
Longitudinal cracks	1754
Loosening	109
Alligator cracking	32
Potholes	28
Settling	5
Rutting	2
Block cracking	3
Total	4074

**Table 3:** The mean value and range of pavement deflection value and PSSI value.

Data Type	Average Value	Range
Pavement deflection/0.01mm	17.65	8.13~30.51
PSSI	93.355	57.63~99.68

**Table 4:** The number of structural damage statistics.

Structural Damage Type	Quantity (Occurrences)
Structural cracks	836
Poor bonding between surface and base layer	99
Poor bonding within base layer	402
Surface loosening	7
Base layer loosening	38
Uneven settling of subgrade	3
Total	1405

**Table 5:** The number and size of structural cracks based on ground penetrating radar detection.

Type	Quantity (Count)	Length Range (m)	Depth Range (cm)	Width Range (mm)
Cracks only in base layer	693	1.5–13	18–54	0–5
Cracks only in surface layer	30	1.5–4.5	0–18	0–4
Cracks in both surface and base	113	1.5–23	0–54	0–6

**Table 6:** Core sample information statistics.

Core Sample Quantity and Proportion		Structural Layer Integrity Evaluation	
		Intact Structural Layer	Severe Damage to Surface or Base Layer
Crack Condition	Cracks penetrating both surface and base layers	0	42 samples, accounting for 58.3%
	Cracks penetrating the surface layer	0	17 samples, accounting for 23.6%
	Cracks existing in both surface and base layers	1 sample, accounting for 1.4%	4 samples, accounting for 5.6%
	Cracks existing in the base layer	3 samples, accounting for 4.2%	5 samples, accounting for 6.9%

**Table 7:** Material parameter table of each structural layer of road.

Layer	Material	Thickness/cm	Dynamic Compression Modulus/MPa	Elastic Modulus /MPa	Damping Coefficient	Density/kg/m^3^
Surface Layer	SMA13- SBS Modified Asphalt	4	8600		0.05	2400
	AC20	6	9200		0.05	2390
	AC25	8	9200		0.05	2380
Base Layer	Cement Stabilized Gravel	18		11,500	0.8	2400
	Cement Stabilized Gravel	18		11,500	0.8	2400
Sub-base Layer	Cement Stabilized Sand-Gravel	18		10,000	0.8	2400
Subgrade	Low Plasticity Clay	—		60	0.4	1800

**Table 8:** Relaxation modulus viscoelastic Prony series of each asphalt mixture.

Relaxation Time/s	SMA13	AC20	AC25
0.0001	0.12734	0.09046	0.07845
0.001	0.17877	0.13667	0.11686
0.01	0.25998	0.22808	0.20089
0.1	0.21919	0.25391	0.24342
1	0.12627	0.19037	0.21453
10	0.04332	0.07047	0.10207
100	0.02001	0.02166	0.03189
1000	0.00473	0.00388	0.00725
10,000	0.00417	0.00251	0.00262
10,0000	0.00082	0.00022	0.00074
Transient shear modulus (20 °C)/MPa	7902	6911	8173

**Table 9:** Deflection test 9 measuring point location distribution.

Sensor Number	D1	D2	D3	D4	D5	D6	D7	D8	D9
Distance/cm	0	20	30	45	60	90	120	150	180

**Table 10:** The width, length, and height of structural cracks in the simulation model.

	Width/mm	1	2	3	4	5	10	15
Length/m								
1.87		√	√	√	√	√	√	√
3.75		√	√	√	√	√	√	√
7.5		√	√	√	√	√	√	√
10.50		√	√	√	√	√	√	√
The location of the structural layer where the crack develops		Cracks develop from the bottom of the base layer upwards. For each width and length, there are four possible development scenarios: extending to the bottom of the lower layer, the bottom of the middle layer, the bottom of the upper layer, and the road surface.						

**Table 11:** Grading standard of pavement structure condition based on structural crack evaluation.

Evaluation Index	Excellent	Good	Medium
PSCI	≥90	≥80, <90	≥70, <80

**Table 12:** Comprehensive Evaluation and Classification of Structural and Surface Cracks.

Evaluation Index	Excellent	Good
SCRR	≥1	<1

**Table 13:** The analytic hierarchy process and entropy weight method combined valuation method.

**Judgment matrix**		**Structural Cracks in the Base Layer**	**Structural Cracks in Both the Surface Layer and the Base Layer**	**Structural Cracks in the Surface Layer**
	Structural cracks existing in the base layer	1	5	7
	Structural cracks existing in both the surface layer and the base layer	1/5	1	3
	Structural cracks existing in the surface layer	1/7	1/3	1
$\mathrm{AHP}\;\mathrm{weight}\;\omega_{1}$		0.2	0.7	0.1
$\mathrm{Entropy}\;\mathrm{weight}\;\omega_{2}$		0.8	0.2	0
$\mathrm{Combined}\;\mathrm{weight}\;\omega_{i}\prime$		1.1	1	0.1

**Table 14:** The cluster center summary.

	N = 2	N = 3	N = 4
Cluster center	0.053	0.017	0.002
	0.607	0.362	0.208
		0.92	0.454
			0.961

**Table 15:** Detect the range of road section evaluation indicators.

Evaluation Indicator	Overall Assessment Result	Percentage of Pavement Sections in Different Evaluation Grades	
		Excellent	Good and Below
Structural crack evaluation index (PSCI)	92.391	75.5%	24.5%
Structural crack reflection ratio (SCRR)	0.340	19.8%	80.2%
Pavement structural strength index (PSSI)	93.355	95.9%	4.1%
Invisible crack rate (ICR) (%)	0.278	82.7%	17.3%

**Table 16:** Detection section classification standard.

Pavement Condition Type	PSSI	PCI	PSCI	SCRR
I	Meets requirements	≥A	≥B	—
II			<B	—
III		<A	≥B	≥C
IV				<C
V			<B	≥C
VI				<C
VII	Does not meet requirements	—		

**Table 17:** Evaluation index standard value reference range.

Indicator Name	PCI	PSCI	SCRR
	A	B	C
Reference Value	87~92	80~90	1

**Table 18:** Standard for grading the intensity of conservation measures.

Strength Grade	Maintenance Measures
P1	Routine Maintenance Measures: Crack sealing, joint sealing, and other routine maintenance measures
P2	Surface Functional Maintenance Measures: Fog seal, microsurfacing, thin overlay, in-situ hot recycling, and grouting
P3	Milling and Overlay with Local Crack Treatment: Milling and repaving the surface layer, or adding an additional layer, with localized crack treatment
P4	Milling and Repaving of Middle and Upper Layers with Local Crack Treatment: Milling and repaving the middle and upper layers, or milling and overlaying one layer with two additional layers
P5	Milling and Repaving of the Surface Layer with Local Crack Treatment: Milling and repaving the surface layer, or milling two layers and adding three layers.
P6	Milling and Repaving of Surface Layer and Base Layer: Milling and re-paving both the surface layer and base layer.

## Data Availability

The original contributions presented in this study are included in the article. Further inquiries can be directed to the corresponding author.
